# The Relationship Between Resting Heart Rate and Cardiovascular Risk Factors Among Adults Aged 18 Years and Above in the Rural Ellisras Population: Ellisras Longitudinal Study

**DOI:** 10.3390/jcdd12110428

**Published:** 2025-10-30

**Authors:** Mohale Gift Maake, Kotsedi Daniel Monyeki, Machoene Derrick Sekgala

**Affiliations:** 1 Department of Physiology and Environmental Health, University of Limpopo, Sovenga 0727, South Africa; 201917159@keyaka.ul.ac.za (M.G.M.); kotsedi.monyeki@ul.ac.za (K.D.M.); 2 Division of Research Capacity Development, South African Medical Research Council, Cape Town 7501, South Africa; 3 Department of Social Sciences, Center for Social Sciences Research (CSSR), University of Cape Town, Robert Leslie Social Science Building 12 University Avenue, Cape Town 7701, South Africa

**Keywords:** resting heart rate, cardiovascular risk factors, rural population, South Africa, Ellisras, cardiometabolic health, gender differences

## Abstract

(1) Background: Resting heart rate (RHR) is an easily measurable cardiovascular risk indicator, yet its relationship with cardiometabolic risk factors remains understudied in rural African populations. This study investigated the association between RHR and cardiovascular risk factors among adults in the rural Ellisras community, South Africa. (2) Methods: A cross-sectional analysis was conducted among 629 participants (306 males, 323 females) aged 18 years and above from the Ellisras Longitudinal Study. Anthropometric measurements, blood pressure, biochemical parameters, and lifestyle factors were assessed. RHR was categorized as normal (60–100 bpm), bradycardia (<60 bpm), or tachycardia (>100 bpm). Statistical analysis included descriptive statistics, chi-square tests, Pearson correlations, and logistic regression to identify predictors of abnormal RHR. (3) Results: The mean age was 25.55 ± 1.97 years, with significant gender differences in cardiovascular parameters. Females had higher RHR (81.78 ± 11.73 vs. 70.36 ± 12.89 bpm, *p* < 0.001), body mass index (BMI) (24.62 vs. 20.67 kg/m^2^, *p* < 0.001), and waist circumference (WC) (81.00 vs. 73.50 cm, *p* < 0.001). Resting heart rate (RHR) distribution varied significantly by gender (*p* < 0.001), with bradycardia more prevalent in males (91.3% vs. 8.7%) and tachycardia in females (75.0% vs. 25.0%). Significant positive correlations were observed between RHR and age (r = 0.105, *p* = 0.009), diastolic blood pressure (DBP) (r = 0.135, *p* < 0.001), fasting blood glucose (FBG) (r = 0.098, *p* = 0.016), total cholesterol (TCHOL) (r = 0.168, *p* < 0.001), LDL-C (r = 0.201, *p* < 0.001), WC (r = 0.169, *p* < 0.001), and sum of 4 skinfolds (bicep, tricep, subscapular and supraspinale) and (r = 0.184, *p* < 0.001). A negative correlation was found with systolic blood pressure (SBP) (r = −0.105, *p* < 0.001). In the logistic regression analysis, participants aged >25 years had significantly lower odds of abnormal RHR (OR = 0.50, 95% CI: 0.26–0.97, *p* = 0.039), after adjusting for confounders. In the fully adjusted model, RHR remained significantly associated with DBP (β = 0.59, *p* < 0.001), LDL-C (β = 2.76, *p* = 0.008), WC (β = 0.10, *p* = 0.012), and triglycerides (TG) (β = 2.78, *p* = 0.002). (4) Conclusions: RHR demonstrates significant associations with multiple cardiovascular risk factors in this rural South African population, with distinct gender-specific patterns. Age emerged as the primary independent predictor of abnormal RHR. These findings suggest that RHR could serve as a valuable, cost-effective screening tool for cardiovascular risk assessment in resource-limited rural settings.

## 1. Introduction

Cardiovascular diseases (CVDs) remain the leading cause of morbidity and mortality worldwide, with disproportionate burden affecting rural populations [1,2]. Recent evidence indicates that rural communities experience higher rates of cardiovascular risk factors including high blood pressure, high cholesterol, diabetes, and heart disease compared to urban areas, with rural individuals demonstrating higher prevalence of traditional risk factors including reduced physical activity, diabetes, obesity, and hypertension [3]. These disparities are particularly pronounced in low- and middle-income countries where rural populations face additional challenges related to healthcare access, socioeconomic factors, and lifestyle transitions [4].

Resting heart rate (RHR) has emerged as a significant and easily measurable cardiovascular risk indicator that provides valuable insights into autonomic nervous system function and overall cardiovascular health [3,5]. RHR predicts both cardiovascular and noncardiovascular death in different populations, though results regarding the association between RHR and specific cardiovascular disease subtypes remain inconsistent [6,7]. Epidemiological studies have demonstrated that individuals with RHR of 60–80 beats/min show increased relative risk for all-cause mortality (RR: 1.12, 95% CI: 1.07–1.17), while those with RHR > 80 beats/min exhibit even higher risk [8]. The clinical significance of RHR extends beyond simple risk stratification, as elevated RHR frequently co-occurs with other cardiovascular risk factors, notably hypertension, atherogenic blood lipid profiles, altered blood glucose and insulin levels, and overweight [7,8].

Rural South African populations represent a unique demographic experiencing rapid epidemiological transitions, where traditional lifestyle patterns intersect with emerging non-communicable disease burdens [9,10]. The Ellisras Longitudinal Study, conducted in the rural village of Ellisras in Limpopo Province, South Africa, has provided valuable insights into cardiovascular risk factors among young adults in this population [11,12]. Previous investigations within this cohort have established important associations between anthropometric indicators of obesity and blood pressure among rural South African populations [13], while other studies have examined patterns of physical activity and cardiovascular disease risk in both urban and rural settings among black South African adults [14,15].

The burden of cardiovascular risk factors in rural African populations is compounded by multiple intersecting challenges. Rural populations are significantly older than urban populations and demonstrate higher rates of diabetes mellitus, obesity, hypertension, and tobacco use [16,17]. Additionally, structural factors including inequities in healthcare access and limited availability of affordable healthy foods contribute to increased cardiovascular disease risk in rural areas [18,19]. These contextual factors necessitate comprehensive understanding of cardiovascular risk markers that can be readily assessed in resource-limited settings.

Despite the established importance of RHR as a cardiovascular risk indicator, limited research has specifically examined its relationship with comprehensive cardiometabolic risk profiles in rural African populations. Previous work in the Ellisras population has identified gaps in cardiovascular disease risk factor knowledge among rural adolescents and young adults, highlighting the need for better characterization of easily measurable risk indicators in this population [12,20]. Furthermore, the high prevalence of overweight and obesity among rural Ellisras females, coupled with dietary risk factors, suggests complex interactions between lifestyle factors and cardiovascular risk that require detailed investigation [11,17].

The relationship between RHR and cardiovascular risk factors may be particularly relevant in rural populations where healthcare resources are limited and early identification of at-risk individuals is crucial for preventive interventions. Understanding these associations can inform the development of cost-effective screening strategies and targeted interventions appropriate for rural healthcare settings. Moreover, the unique demographic and epidemiological characteristics of rural South African populations, including the ongoing nutrition transition and changing physical activity patterns, provide an important context for examining cardiovascular risk relationships. Therefore, this study aims to investigate the relationship between RHR and cardiovascular risk factors among adults aged 18 years and above in the rural Ellisras population.

## 2. Materials and Methods

### 2.1. Study Design and Population

The Ellisras Longitudinal Study (ELS), initiated in 1996, employed a cluster sampling method [11,21]. Full details of the study design and sampling have been described elsewhere [11,21].

The current cross-sectional analysis used ELS samples and included sociodemographic, anthropometric, blood pressure, and biochemical data from 629 participants, randomly selected based on availability of their data and samples. Only participants who were 18 years and older at the time, with complete anthropometric, biochemical, blood pressure, and resting heart rate (RHR) data were included in the analysis. Comparison of basic demographic variables between included and excluded individuals was performed and no significant differences in age or sex distribution were found. The current study has also been ethically approved by the Turfloop Research Ethics Committee (TREC) of the University of Limpopo (TREC/142/2025: PG; date of approval: 18 March 2025).

### 2.2. Anthropometry

All study participants underwent a series of anthropometric measurements (height, weight, skinfolds, and waist circumferences) according to standard procedures of the International Society for the Advancement of Kinanthropometry (ISAK) [22]. Weight was measured using an electronic scale to the nearest 0.1 kg, and height was measured using a Martin anthropometer to the nearest 0.1 cm. Skinfolds (triceps, biceps, subscapular, and supraspinal) were measured using a Slim Guide skinfold calliper, with measurements taken three times and values rounded off to the nearest 0.1 mm. A flexible steel tape was used to measure the waist circumference (WC) to the nearest 0.1 cm, midway between the last rib and the iliac crest, with participants standing. During analysis, waist-to-height ratio (WtHR) was calculated as WC divided by height. Females with a WC ≥ 80 cm and males with a WC ≥ 94 cm were classified as centrally obese [23]. Body mass index (BMI) was calculated as weight (kg) divided by height squared (m^2^). The recommended World Health Organisation (WHO) cut-off points were used to classify underweight (BMI < 18.5 kg/m^2^), normal weight (BMI = 18.5–24.9 kg/m^2^), overweight (BMI = 25.0–29.9 kg/m^2^), and obesity (BMI ≥ 30 kg/m^2^) [24].

### 2.3. Blood Pressure and Resting Heart Rate Measurements

Using an electronic Micronta monitoring kit (RadioShack; Fort Worth, TX, USA; Model No. 98013), at least three blood pressure readings (systolic and diastolic) and heart rates were taken at five-minute intervals after the participant had been seated for 5 min or longer. The device’s electronic infrasonic transducer displayed blood pressure and heart rate concurrently. During the analysis of the results, participants were classified in accordance with the American Heart Association (AHA) 2017 guidelines [25]. Blood pressure was classified as: normal (<120/<80 mmHg), elevated (120–129/<80 mmHg), stage 1 hypertension (130–139/80–89 mmHg), and stage 2 hypertension (≥140/≥90 mmHg).

Resting heart rate (RHR) categories were: normal (60–100 bpm), bradycardia (<60 bpm), and tachycardia (>100 bpm) [25]. We note that the recently published 2025 AHA/ACC guideline on blood pressure and cardiovascular risk management [26] reaffirms the importance of standardized blood pressure and heart rate measurement, and provides updated thresholds for clinical practice that are consistent with the framework applied in our study.

### 2.4. Fasting Blood Glucose

Fasting blood samples were collected into 4 mL grey-top Vacutainer® tubes containing sodium fluoride/potassium oxalate as anticoagulant (Becton, Dickinson and Company, Franklin Lakes, NJ, USA). Participants fasted for 8 to 10 h before collection, in accordance with the ELS protocol, as per the consent form. Samples were then placed in a cooler box with ice (0–8 °C) on site prior to analysis. During analysis, fasting blood glucose (FBG) was measured using the glucose oxidase method, where glucose oxidase will catalyse the oxidation of glucose to gluconic acid, producing hydrogen peroxide. This was further broken down by peroxidase, resulting in a colour change proportional to the glucose concentration. The measurement was performed on a Beckman LX20^®^ auto analyser (Beckman Coulter, Fullerton, CA, USA). FBG levels were then categorized as: below 3.9 mmol/L as low, 3.9–5.6 mmol/L as normal, 5.6–6.9 mmol/L as prediabetic, and above 7 mmol/L as diabetic [27].

### 2.5. Lipid Profile

At the South African Medical Research Council (SAMRC) in Cape Town, South Africa, fasting blood samples have been centrifuged at 2500 rpm for 15 min, in 4 mL gold top vacutainer tubes (vacutainer BD^TM^) containing serum separator gel, to obtain plasma. The plasma has been stored at −80 °C prior to measuring of the lipid profiles. Total cholesterol (TCHOL), triglycerides (TG), and high-density lipoprotein cholesterol (HDL-C) were measured using enzymatic assay kits on a Beckman LX20^®^ auto analyser. During measuring of the lipid profiles, Low-density lipoprotein cholesterol (LDL-C) was calculated using the Friedewald equation: [LDL-C] = [TCHOL] − [HDL-C] − [TG]/5 [28]. The abnormal level cut-off points (measured in millimoles per litre of blood—mmol/L) for each of the four standard tests in a lipid panel were as follows: TCHOL > 5.1 mmol/L was considered abnormal [29]; HDL-C < 1.0 mmol/L (for males) and HDL-C ˂ 1.2 mmol/L (for female) were considered abnormal [23]; LDL-C > 3.0 mmol/L was considered abnormal [29]; TG > 1.7 mmol/L was considered abnormal [23].

### 2.6. Tobacco Use

The questionnaire used in this study is based on questions from the Birth-To-Ten Study [30], the Amsterdam Growth and Health Longitudinal Study [31] and the Global Youth Tobacco Study [32], covering important issues relating to tobacco use. The questionnaire includes procedures ensuring face, content, and criterion validity. It was translated into the local languages (Sepedi and Setswana) and then back translated into English before use. The questionnaire comprised two sections: demographic questions (age, gender, educational level) and questions assessing tobacco use practices. Participants were asked if they currently use tobacco (yes/no). A negative answer (no) classified them as abstainers. A positive answer (yes) led to further questions about the type of tobacco used (cigarettes, pipes, hand-rolled, smokeless products like snuff and chewing). Participants were also asked about their daily use of each type. To quantify total tobacco exposure, self-reported amounts of cigarettes, pipes, and snuff were converted into a scale using the approximate amount of tobacco contained in each form. One pipe was converted into a representation of 2 cigarettes equivalent. Tobacco use was categorized as “<10 cigarettes equivalent per day,” “10–20 cigarettes per day,” and “>20 cigarettes equivalent per day.” Cigarette smoking was defined as smoking at least one cigarette per day for at least one year [33]. Total tobacco use was quantified as pack-years, defined as the number of packs of cigarettes or cigarette equivalents per day (1 pack = 20 cigarettes) multiplied by the number of years smoked [33].

### 2.7. Alcohol Consumption

Alcohol intake was assessed using a structured questionnaire following the standard proposed by the UK Chief Medical Officer’s low-risk drinking guidelines [34]. The guidelines advise that it is safest not to drink more than 14 units of alcohol per week on a regular basis. Regular drinking of more than 14 units per week categorizes individuals as high risk for medical conditions like cardiovascular disorders [34]. Consumption was converted into units based on the alcohol type and alcohol percentage. 1 unit represented wine (13%, 76 mL), whisky (40%, 25 mL), beer (4%, 250 mL) or homebrew (5.25%, 250 mL). Participants were asked about their weekly consumption of each type of alcohol, which were then categorized into light drinkers (1–3 units per week), moderate drinkers (>3 to 6 units per week for females and >3 to 14 units per week for males), and heavy drinkers (>6 units per week for females and >14 units per week for males) [35]. Communal drinkers (occasional/social drinkers) were those who drink in low-risk patterns without intent to become intoxicated [36].

### 2.8. Physical Activity

The International Physical Activity Questionnaire was found to be reliable and valid [37]. This questionnaire was used to record data on work, leisure, and travel activities during both weekdays and weekends. The scores for these activities were obtained by multiplying the number of reported activities by the number of days engaged in those activities per week. The total physical activity score was calculated by summing the types of activities for each participant. The durations of these activities were also averaged to give a mean duration for moderate to vigorous physical activities during weekdays and weekends. Metabolic equivalents (METs) express the energy cost of physical activities as a multiple of the resting metabolic rate and yield score in MET-minutes. MET-minutes were obtained by multiplying the MET score (8 for vigorous and 4 for moderate activity) by the minutes performed [38].

### 2.9. Quality Control

A pilot study was conducted prior to the main data collection, during which selected ELS fieldworkers received training in anthropometric measurement following the ISAK standard procedures [22]. This training ensured that the fieldworkers could competently measure the anthropometric variables. Inter- and intra-tester technical errors of measurement for WC, hip circumference, and height were assessed. All instruments were calibrated before data collection. The reliability and validity procedures for this study have been reported in detail elsewhere [39,40].

### 2.10. Statistical Analysis

Data analysis was conducted using SPSS version 28.0 (IBM Corporation, Armonk, NY, USA). Descriptive statistics were calculated for all variables, with continuous variables presented as means ± standard deviations for normally distributed data or medians with interquartile ranges (IQR) for non-normally distributed data. Categorical variables were presented as frequencies and percentages. The normality of continuous variables was assessed using the Shapiro–Wilk test and visual inspection of histograms and Q-Q plots. Independent samples t-tests were used to compare normally distributed continuous variables between groups, while Mann–Whitney U tests were employed for non-normally distributed variables. Chi-square tests were used to examine associations between categorical variables. Pearson correlation and linear regression (adjusting for the continuous variables as confounders) coefficients were calculated to assess linear relationships between RHR and continuous cardiometabolic variables. For the adjusted linear regression, we included age, average SBP, average DBP, FBG, HDL-C, LDL-C, TCHOL/HDL-C ratio, WC, sum of 4 skinfolds, and TG. WtHR was excluded due to collinearity with WC, which showed stronger associations. Variance inflation factors (all < 5) confirmed the absence of problematic multicollinearity. The strength of correlations was interpreted according to Cohen’s guidelines: r = 0.10–0.29 (small), r = 0.30–0.49 (medium), and r ≥ 0.50 (large effect sizes) [41]. Resting heart rate (RHR) was categorized into normal (60–100 beats per minute) and abnormal (bradycardia: <60 bpm; tachycardia: >100 bpm) based on established clinical criteria. Logistic regression analysis was performed to identify predictors of abnormal RHR. Univariate logistic regression was initially conducted for each potential predictor variable, followed by multivariate analysis including all variables with *p* < 0.25 in univariate analysis or those deemed clinically important. Model assumptions for logistic regression were verified, including linearity of continuous variables with the logit, absence of multicollinearity (variance inflation factor < 5), and adequate sample size relative to the number of predictor variables. For multivariate logistic regression, variables with *p* < 0.25 in univariate analysis or variables deemed clinically important were entered into the model. To minimize collinearity, WC and WtHR were not included simultaneously; WC was retained as it demonstrated stronger associations in preliminary analyses, while WtHR was excluded and vice versa. The final multivariate model included age group, physical activity, BMI category, WC (central obesity), blood pressure category, HDL-C status, LDL-C status, TG status, TCHOL status, LDL/HDL-C ratio, TCHOL/HDL-C ratio, sum of 4 skinfolds, and FBG category. Variance inflation factors (all < 5) confirmed absence of problematic multicollinearity. Sex was excluded from the final multivariate regression models because of multicollinearity with RHR categories, as confirmed by variance inflation factors (>5 when sex was included). The Hosmer-Lemeshow goodness-of-fit test was used to assess model fit [42]. Odds ratios (OR) and 95% confidence intervals (CI) were calculated for all predictor variables. All statistical tests were two-tailed, and statistical significance was set at *p* < 0.05. Bonferroni correction was applied to multiple comparisons where appropriate to control for Type I error inflation [43].

## 3. Results

A total of 629 participants were included in this study, comprising 306 males (48.6%) and 323 females (51.4%). The overall mean age was 25.55 ± 1.97 years, with no statistically significant difference between males and females (25.45 ± 1.93 vs. 25.65 ± 2.01 years, *p* = 0.196). Significant gender differences were observed across multiple anthropometric and cardiovascular parameters (Table 1). Males demonstrated significantly greater height (173.71 ± 13.54 vs. 163.07 ± 10.20 cm, *p* < 0.001) and systolic blood pressure (SBP) (125.91 ± 12.48 vs. 114.11 ± 10.83 mmHg, *p* < 0.001) compared to females. Conversely, females exhibited significantly higher RHR (81.78 ± 11.73 vs. 70.36 ± 12.89 bpm, *p* < 0.001), TCHOL (4.26 ± 1.12 vs. 4.03 ± 0.94 mmol/L, *p* = 0.004), and low-density lipoprotein cholesterol (LDL-C) (2.98 ± 0.95 vs. 2.61 ± 0.81 mmol/L, *p* < 0.001). Males showed significantly higher high-density lipoprotein cholesterol (HDL-C) levels (1.20 ± 0.37 vs. 1.09 ± 0.30 mmol/L, *p* < 0.001). Regarding anthropometric measurements, females had significantly higher median BMI (24.62 vs. 20.67 kg/m^2^, *p* < 0.001), WC (81.00 vs. 73.50 cm, *p* < 0.001), hip circumference (102.90 vs. 90.80 cm, *p* < 0.001), and all skinfold measurements (all *p* < 0.001). The WtHR was also significantly higher in females (0.50 vs. 0.41, *p* < 0.001).

Table 2 presents the distribution of categorical variables by gender and RHR status. Significant gender differences were observed in employment status (*p* = 0.015), with males more likely to be laborers (60.0%) compared to females (40.0%). Physical activity levels also differed significantly between genders (*p* = 0.003), with males showing higher rates of vigorous activity (64.6% vs. 35.4%). The distribution of RHR categories showed marked gender differences (*p* < 0.001). Bradycardia was predominantly observed in males (91.3% vs. 8.7%), while tachycardia was more prevalent in females (75.0% vs. 25.0%). Normal RHR was more common in females (55.5% vs. 44.5%). Several cardiovascular risk factors showed significant associations with abnormal RHR. Blood pressure categories were significantly associated with RHR status (*p* = 0.026), with elevated blood pressure showing higher rates of abnormal RHR (22.9% vs. 13.8% for normal blood pressure). Body Mass Index (BMI) categories demonstrated a significant relationship with RHR (*p* = 0.002), where underweight individuals had the highest prevalence of abnormal RHR (24.4%). Physical activity levels were significantly associated with RHR status (*p* = 0.038), with moderately active individuals showing the highest rate of abnormal RHR (28.6%). Central obesity, as defined by WC, was significantly associated with RHR status (*p* = 0.001), with centrally obese individuals having lower rates of abnormal RHR (8.2% vs. 19.0%).

Pearson correlation analysis revealed several significant associations between RHR and cardiometabolic variables (Table 3). Resting Heart Rate (RHR) showed statistically significant positive correlations with age (r = 0.105, *p* = 0.009), DBP (r = 0.135, *p* < 0.001), FBG (r = 0.098, *p* = 0.016), and various lipid parameters including TCHOL (r = 0.168, *p* < 0.001), LDL-C (r = 0.201, *p* < 0.001), LDL-C/HDL-C ratio (r = 0.181, *p* < 0.001), and TCHOL/HDL-C ratio (r = 0.187, *p* < 0.001). Strong positive correlations were also observed between RHR and anthropometric measures, including WC (r = 0.169, *p* < 0.001), WtHR (r = 0.146, *p* < 0.001), and sum of for 4 skinfolds (bicep, tricep, subscapular and supraspinale) (r = 0.184, *p* < 0.001). Triglycerides (TG) showed a moderate positive correlation with RHR (r = 0.124, *p* = 0.002). Notably, a significant negative correlation was found between RHR and SBP (r = −0.105, *p* < 0.001). No statistically significant correlations were observed between RHR and HDL-C (r = −0.064, *p* = 0.108) or BMI (r = 0.037, *p* = 0.350).

Linear regression analysis revealed significant associations between RHR and multiple cardiometabolic variables in both unadjusted and adjusted models (Table 4). In the fully adjusted linear regression model (Model 2), average DBP (β = 0.50, *p* < 0.001), LDL-C (β = 2.76, *p* = 0.008), WC (β = 0.10, *p* = 0.012), sum of four skinfolds (β = 0.06, *p* = 0.011), TG (β = 2.78, *p* = 0.002), and the TCHOL/HDL-C ratio (β = 0.93, *p* = 0.04) remained significantly associated with RHR. Age did not retain statistical significance after adjustment (β = 0.39, *p* = 0.141), and FBG was borderline (β = 0.78, *p* = 0.050).

Univariate logistic regression analysis identified several significant predictors of abnormal RHR (Table 5). Participants aged >25 years had significantly lower odds of abnormal RHR compared to those aged 18–25 years (OR = 0.58, 95% CI: 0.38–0.89, *p* = 0.012). Moderate physical activity was associated with decreased odds of abnormal RHR (OR = 0.26, 95% CI: 1.22–5.47, *p* = 0.013). Among BMI categories, overweight individuals showed significantly reduced odds of abnormal RHR compared to normal weight participants (OR = 0.32, 95% CI: 0.14–0.73, *p* = 0.007). Participants who were not centrally obese had lower odds of abnormal RHR (OR = 0.38, 95% CI: 0.21–0.70, *p* = 0.002). Elevated blood pressure was associated with increased odds of abnormal RHR (OR = 1.85, 95% CI: 1.13–3.04, *p* = 0.014). Abnormal HDL-C levels were associated with significantly lower odds of abnormal RHR (OR = 0.52, 95% CI: 0.34–0.80, *p* = 0.003). The sum of 4 skinfolds showed a small but significant protective effect (OR = 0.98, 95% CI: 0.97–1.00, *p* = 0.005). In the fully adjusted multivariate model (Model 2), only age group remained statistically significant. Participants aged >25 years maintained significantly lower odds of abnormal RHR after adjusting for all covariates (OR = 0.50, 95% CI: 0.26–0.97, *p* = 0.039). All other variables lost statistical significance in the adjusted model, suggesting potential confounding effects or multicollinearity among the predictors. The multivariate analysis indicates that age is the primary independent predictor of abnormal RHR in this population, with older participants having approximately 50% lower odds of experiencing abnormal RHR patterns.

## 4. Discussion

This study provides the first comprehensive examination of the relationship between RHR and cardiovascular risk factors in a rural South African population. Our findings reveal several important patterns: significant gender differences in RHR distribution and cardiovascular risk profiles, positive correlations between RHR and multiple cardiometabolic parameters, and age as the primary independent predictor of abnormal RHR. The most striking finding was the marked gender disparity in RHR categories, with bradycardia predominantly affecting males and tachycardia more prevalent in females. Additionally, RHR demonstrated significant positive correlations with age, DBP, FBG, lipid parameters, and anthropometric measures, while showing a negative correlation with SBP.

Our findings of higher RHR in females compared to males align with established literature demonstrating consistent gender differences in cardiac autonomic function [44,45]. Previous meta-analyses have reported inconsistent findings regarding gender differences in heart rate variability, but recent genetic studies have identified sex-specific effects underlying RHR and associated cardiovascular disease risk [44]. The magnitude of the gender difference observed in our study (approximately 11 bpm) is comparable to differences reported in other populations, suggesting that the underlying physiological mechanisms are consistent across diverse ethnic groups [46]. Studies examining gender-related differences in beat-to-beat heart rate dynamics have proposed that women may have more complex cardiac autonomic regulation than men, which could partially explain the higher prevalence of tachycardia in females observed in our study. However, the predominance of bradycardia in males in our rural population is notable and may reflect higher levels of physical activity or different autonomic nervous system responses in this demographic [47]. These findings highlight the importance of examining sex-specific associations, as cardiovascular risk factor expression and autonomic patterns differ between males and females, particularly in transitional stages such as menopause.

The positive correlations between RHR and cardiometabolic risk factors observed in our study are consistent with findings from large epidemiological studies. It should be noted that while several of the observed correlations were statistically significant, the effect sizes were small, reflecting weak associations. This is not unexpected given the young age of our sample, in which cardiovascular risk factors may not yet strongly expressed, but such early signals may still hold prognostic value for long term risk trajectories. Recent meta-analyses have confirmed RHR as an independent predictor of cardiovascular and all-cause mortality in both men and women, supporting our findings of significant associations with multiple risk factors [6,8]. The correlation and associations between RHR and lipid parameters, anthropometric measures, and blood pressure is particularly noteworthy, as it suggests a potential mechanistic link between sympathetic nervous system activation and atherogenic lipid profiles. The maintained significance of multiple risk factors supports the potential utility of RHR as a simple screening tool that captures diverse aspects of cardiometabolic risk in rural populations. Interestingly, underweight participants showed higher odds of abnormal RHR, which may reflect malnutrition-related autonomic imbalance. Conversely, central obesity appeared inversely associated with abnormal RHR, a paradoxical finding likely driven by the predominance of bradycardia among males, who also had lower central obesity prevalence. This highlights the complexity of interpreting crude associations in narrowly distributed young populations.

Interestingly, our finding of a negative correlation between RHR and SBP contrasts with some previous studies that have reported positive associations [48,49]. This inverse relationship may reflect the younger age of our population and the predominance of bradycardia in males, who typically have higher SBP. A recent Chinese study found that low baseline heart rate (<65 bpm) was associated with higher cardiovascular disease risk, which may partially explain our findings [50]. Furthermore, the negative association persisted in our adjusted linear regression model, suggesting this relationship is independent of other cardiovascular risk factors. This finding may indicate that in younger populations with lower baseline cardiovascular risk, the traditional positive RHR-blood pressure relationship may be altered by factors such as physical fitness, autonomic nervous system maturation, or population-specific genetic variations that warrant further investigation in longitudinal studies. Beyond biological and lifestyle factors, psychosocial stressors including occupational strain, financial insecurity, and work-related stress are known to influence autonomic function and RHR [51]. Although we did not capture these variables in our dataset, they represent important contextual determinants in rural populations and may partially explain inter-individual variability in RHR.

We would like to acknowledge the study limitation includes cross-sectional design which precludes causal inference, and longitudinal follow-up would be necessary to establish temporal relationships between RHR and cardiovascular outcomes. The study population is relatively young (mean-age 25.55 years), which may limit generalizability to older adults who typically have higher cardiovascular risk. Additionally, the rural South African context may limit external validity to other populations or settings. Some important confounding variables were not measured, including medication (particularly beta-blockers or other cardiovascular medications), caffeine consumption, and stress levels, all of which can significantly influence RHR. The definition of physical activity categories may not have captured the full spectrum of activity levels in this rural population, where occupational physical activity may be substantial but not formally categorized. Furthermore, single-time-point measurements of RHR may not reflect long-term patterns, as RHR can vary with time of day, season, and various physiological and environmental factors [52]. The relatively narrow age range also limits our ability to examine age-related changes in RHR-cardiovascular risk relationships across the adult lifespan. Furthermore, the use of self-reported questionnaires to assess behavioral factors is associated with a degree of subjectivity and potential recall bias, which may have influenced the accuracy of lifestyle-related variables such as physical activity and smoking. Intrinsic heart rate was not assessed, which is an independent predictor of pulse wave velocity, related to the risk of cardiovascular events.

## 5. Conclusions

This study demonstrates that RHR is significantly associated with multiple cardiovascular risk factors in a rural South African population, with distinct gender-specific patterns. Age emerged as the primary independent predictor of abnormal RHR, while significant correlations were observed with various cardiometabolic parameters. These findings support the potential utility of RHR as a simple, cost-effective screening tool for cardiovascular risk assessment in resource-limited rural settings. The marked gender differences observed highlight the need for gender-specific approaches to cardiovascular risk assessment in this population. Future longitudinal studies are needed to establish causal relationships and evaluate the prognostic value of RHR in predicting cardiovascular outcomes in rural African populations.

## Figures and Tables

**Table 1:** Baseline Characteristics of Participants by Gender.

	Total (*n* = 629)	Male (*n* = 306)	Female (*n* = 323)	*p*-Value
	Mean ± SD	Mean ± SD	Mean ± SD	
Age (years)	25.55 ± 1.97	25.45 ± 1.93	25.65 ± 2.01	0.196
Height	168.25 ± 13.07	173.71 ± 13.54	163.07 ± 10.20	<0.001
RHR	76.22 ± 13.56	70.36 ± 12.89	81.78 ± 11.73	<0.001
SBP	119.85 ± 13.06	125.91 ± 12.48	114.11 ± 10.83	<0.001
DBP	70.21 ± 9.85	71.44 ± 10.24	69.06 ± 9.33	0.002
FBG	5.54 ± 1.28	5.46 ± 0.87	5.62 ± 1.57	0.127
TCHOL	4.15 ± 1.04	4.03 ± 0.94	4.26 ± 1.12	0.004
HDL-C	1.15 ± 0.34	1.20 ± 0.37	1.09 ± 0.30	<0.001
LDL-C	2.80 ± 0.90	2.61 ± 0.81	2.98 ± 0.95	<0.001
LDL-C/HDL-C_Ratio	2.61 ± 1.14	2.35 ± 1.22	2.85 ± 1.00	<0.001
TCHOL/HDL-C_Ratio	3.80 ± 1.19	3.54 ± 1.28	4.03 ± 1.05	<0.001
BMI (Median (IQR))	22.16 (6.68)	20.67 (4.33)	24.62 (9.22)	<0.001
Weight (Median (IQR))	64.60 (17.55)	63.60 (13.45)	65.40 (22.40)	0.044
Hip circumference (Median (IQR))	95.00 (16.50)	90.80 (10.28)	102.90 (19.00)	<0.001
WC (Median (IQR))	76.00 (16.60)	73.50 (11.00)	81.00 (21.00)	<0.001
Triceps skinfold (Median (IQR))	7.00 (8.67)	5.00 (4.71)	12.17 (11.33)	<0.001
Biceps skinfold (Median (IQR))	5.00 (6.50)	3.00 (2.17)	9.00 (9.00)	<0.001
Subscapular skinfold (Median (IQR))	9.00 (5.75)	7.92 (3.17)	11.00 (7.67)	<0.001
Supraspinale skinfold (Median (IQR))	7.00 (8.00)	4.00 (3.17)	11.00 (9.00)	<0.001
Medialcalf skinfold (Median (IQR))	9.67 (9.92)	5.17 (5.00)	14.67 (9.18)	<0.001
WtHR (Median (IQR))	0.45 (0.12)	0.41 (0.07)	0.50 (0.13)	<0.001
TG (Median (IQR))	0.83 (0.57)	0.87 (0.57)	0.78 (0.58)	0.059

Abbreviations: SBP: Systolic Blood Pressure; DBP: Diastolic Blood Pressure; FBG: Fasting Blood Glucose; TCHOL: Total Cholesterol; HDL-C: High Density Lipoprotein Cholesterol; LDL-C: Low Density Lipoprotein Cholesterol; BMI: Body Mass Index; WC: Waist Circumference; WtHR: Waist-to-height ratio; TG: Triglyceride; IQR: Inter Quartile Range.

**Table 2:** Distribution of Categorical Variables by Gender and Resting Heart Rate.

		Males (*n* = 306)	Females (323)	*p*-Value	Normal RHR	Abnormal RHR	*p*-Value
		*n* (%)	*n* (%)	0.268	*n* (%)	*n* (%)	
Age-group	18–25	323.93 (51.5)	305.07 (48.5)		499 (79.3)	130 (20.7)	0.012
	>25	295 (46.9)	334 (53.1)		547 (86.9)	82 (13.1)	
Education level	Primary	184 (48.4)	197 (51.6)	0.224	320 (83.9)	61 (16.1)	0.870
	Secondary	173 (45.4)	208 (54.6)		322 (84.5)	59 (15.5)	
	Tertiary	115 (30.3)	266 (69.7)		335 (87.9)	46 (12.1)	
Employment status	Labourer	229 (60.0)	152 (40.0)	0.015	319 (83.6)	62 (16.4)	0.116
	Professional	200 (52.6)	181 (47.4)		281 (73.7)	100 (26.3)	
	Unemployed	154 (40.3)	227 (59.7)		330 (86.5)	51 (13.5)	
RHR status	Bradycardia	574 (91.3)	55 (8.7)	<0.001			
	Normal	280 (44.5)	349 (55.5)				
	Tachycardia	157 (25.0)	472 (75.0)				
Blood pressure	Normal	177 (28.2)	452 (71.8)	<0.001	542 (86.2)	87 (13.8)	0.026
	Elevated	428 (68.0)	201 (32.0)		485 (77.1)	144 (22.9)	
	Stage 1 hypertension	413 (65.7)	216 (34.3)		561 (89.2)	68 (10.8)	
	Stage 2 hypertension	514 (81.8)	115 (18.2)		503 (80.0)	126 (20.0)	
FBG	Low	254 (42.1)	350 (57.9)	0.505	477 (78.9)	127 (21.1)	0.159
	Normal	312 (51.7)	292 (48.3)		500 (82.8)	104 (17.2)	
	Pre-diabetic	283 (46.8)	321 (53.2)		532 (88.0)	72 (12.0)	
	Diabetic	256 (42.4)	348 (57.6)		458 (75.8)	146 (24.2)	
TCHOL status	Normal	325 (51.6)	277 (48.4)	<0.001	526 (83.7)	103 (16.3)	0.754
	Abnormal	109 (33.0)	520 (67.0)		535 (85.0)	94 (15.0)	
HDL-C	Normal	247 (75.0)	382 (25.0)	<0.001	498 (79.2)	131 (20.8)	0.003
	Abnormal	166 (26.4)	463 (73.6)		548 (88.0)	81 (12.0)	
LDL-C	Normal	344 (54.7)	285 (45.3)	<0.001	530 (84.2)	99 (15.8)	0.820
	Abnormal	234 (37.2)	395 (62.8)		525 (83.5)	104 (16.5)	
LDL-C/HDL-C ratio	Ideal (≤2.5)	379 (60.3)	250 (39.7)	<0.001	527 (83.8)	102 (16.3)	0.893
	Elevated risk (>2.5)	230 (36.6)	399 (63.4)		529 (84.1)	100 (15.9)	
TCHOL/HDL-C ratio	Acceptable (<5)	325 (51.6)	304 (48.4)	<0.001	525 (83.5)	104 (16.5)	0.410
	Higher risk (≥5)	160 (25.4)	469 (74.6)		549 (87.3)	80 (12.7)	
BMI category	Underweight	433 (68.9)	169 (31.1)	<0.001	476 (75.6)	153 (24.4)	0.002
	Normal	372 (59.1)	257 (40.9)		515 (81.8)	114 (18.2)	
	Overweight	175 (27.9)	454 (72.1)		587 (93.3)	42 (6.7)	
	Obese	92 (14.7)	537 (85.3)		563 (89.5)	66 (10.5)	
Physical activeness	Low	151 (39.6)	230 (60.1)	0.003	330 (86.6)	51 (13.4)	0.038
	Moderate	200 (52.4)	181 (47.6)		272 (71.4)	109 (28.6)	
	Vigorous	246 (64.6)	165 (35.4)		56 (85.4)	325 (14.6)	
WC	Not centrally obese	418 (66.4)	211 (33.4)	<0.001	509 (81.0)	120 (19.0)	0.001
	Centrally obese	0 (0.0)	629 (100)		577 (91.8)	52 (8.2)	
TG	Normal	303 (48.1)	326 (51.9)	0.448	530 (84.3)	99 (15.7)	0.456
	Abnormal	335 (53.2)	294 (46.8)		507 (80.6)	122 (19.4)	
WtHR	Normal	306 (48.6)	323 (51.4)	0.610	524 (83.8)	105 (16.2)	0.326
	Elevated risk	377 (60.0)	252 (40.0)		629 (100.0)	0 (0.0)	
Number of cigarettes	Communal smoking	293 (76.9)	88 (23.1)	<0.001	293 (76.9)	88 (23.1)	0.216
	Less than 10 cigarettes a day	349 (91.7)	32 (8.3)		254 (66.7)	27 (33.3)	
	More than 20 cigarettes a day	354 (92.9)	27 (7.1)		299 (78.6)	82 (21.4)	

Abbreviations: RHR: Resting Heart Rate; FBG: Fasting Blood Glucose; TCHOL: Total Cholesterol; HDL-C: High Density Lipoprotein Cholesterol; LDL-C: Low Density Lipoprotein Cholesterol; BMI: Body Mass Index; WC: Waist Circumference; WtHR: Waist-to-height ratio; TG: Triglyceride.

**Table 3:** Correlation Between Resting Heart Rate and Cardiometabolic Risk Factors.

Variable	Resting Heart Rate	
	Coefficient	*p*-Value
Age	0.105	0.009
Average SBP	−0.105	<0.001
Average DBP	0.135	<0.001
FBG	0.098	0.016
TCHOL	0.168	<0.001
HDL-C	−0.064	0.108
LDL-C	0.201	<0.001
LDL/HDL-C Ratio	0.181	<0.001
TCHOL/HDL-C Ratio	0.187	<0.001
BMI	0.037	0.350
WC	0.169	<0.001
WtHR	0.146	<0.001
TG	0.124	0.002
Sum of 4 skinfolds	0.184	<0.001

Abbreviations: SBP: Systolic Blood Pressure; DBP: Diastolic Blood Pressure; FBG: Fasting Blood Glucose; TCHOL: Total Cholesterol; HDL-C: High Density Lipoprotein Cholesterol; LDL-C: Low Density Lipoprotein Cholesterol; BMI: Body Mass Index; WC: Waist Circumference; WtHR: Waist-to-height ratio; TG: Triglyceride.

**Table 4:** Linear Regression Between Resting Heart Rate and Cardiometabolic Risk Factors.

Cardiometabolic Risk Factors	Unstandardized Coefficients	*p*-Value	Unstandardized Coefficients	*p*-Value
	β (95% CI)		β (95% CI)	
	Model 1 (Un-Adjusted)		Model 2 (Adjusted)	
Age	0.72 (0.18–1.26)	0.009	0.39 (−0.13–0.898)	0.141
Average SBP	−0.17 (−0.25–−0.90)	<0.001	−0.38 (−0.48–−0.28)	<0.001
Average DBP	0.19 (0.08–0.29)	<0.001	0.50 (0.37–0.64)	<0.001
FBG	1.03 (0.19–1.86)	0.016	0.78 (0.00–1.56)	0.050
HDL-C	−2.57 (−5.70–0.56)	0.108	−2.77 (−7.42–1.88)	0.243
LDL-C	3.02 (1.86–4.17)	<0.001	2.76 (0.72–4.80)	0.008
LDL-/HDL-C Ratio	2.15 (1.24–3.07)	<0.001	−0.54 (−2.35–1.28)	0.561
TCHOL/HDL-C Ratio	2.13 (1.25–3.01)	<0.001	0.93 (0.06–1.81)	0.040
WtHR	17.58 (8.27–26.89)	<0.001	−1.06 (−13.77–11.64)	0.870
WC	0.18 (0.10–0.26)	<0.001	0.10 (−0.03–0.23)	0.120
BMI	0.04 (−0.04–0.11)	0.350	−0.28 (−0.10–0.05)	0.462
Sum of 4 Skinfolds	0.10 (0.06–0.15)	<0.001	0.06 (0.01–0.10)	0.011
TG	2.87 (1.07–4.68)	0.002	2.78 (1.02–4.54)	0.002

Abbreviations: SBP: Systolic Blood Pressure; DBP: Diastolic Blood Pressure; FBG: Fasting Blood Glucose; TCHOL: Total Cholesterol; HDL-C: High Density Lipoprotein Cholesterol; LDL-C: Low Density Lipoprotein Cholesterol; BMI: Body Mass Index; WC: Waist Circumference; WtHR: Waist-to-height ratio; TG: Triglyceride. Model 2 (Adjusted): mutually adjusted for age, average SBP, average DBP, FBG, HDL-C, LDL-C, TCHOL/HDL-C ratio, WC, sum of 4 skinfolds, and TG. WC and WtHR were not entered simultaneously due to collinearity; WC was retained and WtHR was excluded, and vice versa. Coefficients shown are unstandardized β (95% CI).

**Table 5:** Logistic Regression For Predictors of Abnormal Resting Heart Rate.

Predictor Variable	Crude OR (95% CI)	*p*-Value	Adjusted OR (95% CI)	*p*-Value
	Model 1 (Unadjusted)		Model 2 (Adjusted)	
Age group (18–25)	Reference			
Age group (>25)	0.58 (0.38–0.89)	0.012	0.50 (0.26–0.97)	0.039
Do you use tobacco? (‘yes’)	Reference			
Do you use tobacco? (‘No’)	0.66 (0.34–1.32)	0.242	0.86 (0.22–3.35)	0.829
Number of cigarettes (Communal smoking)	Reference			
Less than 10 cigarettes a day	1.67 (0.29–9.71)	0.570	2.09 (0.28–15.41)	0.468
More than 20 cigarettes a day	0.91 (0.15–5.58)	0.918	1.18 (0.14–9.70)	0.877
Physical activeness (Not active)	Reference			
Moderately active	0.26 (1.22–5.47)	0.013	0.21 (0.89–5.03)	0.088
Vigorous activity	1.10 (0.46–2.63)	0.825	0.82 (0.31–2.18)	0.683
Risky alcohol intake? (‘yes’)	Reference			
Risky alcohol intake? (‘No’)	0.84 (0.46–1.53)	0.575	1.02 (0.55–1.92)	0.942
BMI (Normal weight)	Reference			
Underweight	1.45 (0.83–2.52)	0.188	1.24 (0.54–2.84)	0.614
Overweight	0.32 (0.14–0.73)	0.007	0.37 (0.07–1.94)	0.240
Obese	0.53 (0.26–1.07)	0.078	1.84 (0.39–8.76)	0.442
WC (Centrally obese)	Reference			
WC (Not centrally obese)	0.38 (0.21–0.70)	0.002	0.72 (0.18–2.97)	0.650
WtHR risk (Normal)	Reference			
WtHR risk (Elevated risk)	0.06 (0.00–0.80)	0.033	0.62 (0.03–11.71)	0.753
Blood Pressure (Normal Blood pressure)	Reference			
Elevated Blood pressure	1.85 (1.13–3.04)	0.014	1.81 (0.82–3.96)	0.140
Stage 1 hypertension	0.76 (0.37–1.52)	0.434	1.05 (0.40–2.81)	0.916
Stage 2 hypertension	1.56 (0.75–3.25)	0.233	0.97 (0.33–2.88)	0.953
HDL-C (Normal)	Reference			
HDL-C (Abnormal)	0.52 (0.34–0.80)	0.003	0.45 (0.20–1.02)	0.057
LDL-C (Normal)	Reference			
LDL-C (Abnormal)	1.05 (0.68–1.64)	0.820	0.82 (0.32–2.10)	0.671
TG (Normal)	Reference			
TG (Abnormal)	1.29 (0.66–2.52)	0.457	1.21 (0.42–3.46)	0.727
TCHOL (Normal)	Reference			
TCHOL (Abnormal)	0.91 (0.50–1.65)	0.754	1.05 (0.36–3.05)	0.928
LDL-C/HDL-C ratio risk (Ideal)	Reference			
LDL-C/HDL-C ratio risk (Elevated risk)	0.90 (0.72–1.12)	0.331	1.93 (0.86–4.34)	0.109
TCHOL/HDL-C ratio risk (Ideal)	Reference			
TCHOL/HDL-C ratio risk (Elevated risk)	0.91 (0.74–1.12)	0.364	1.45 (0.45–4.71)	0.539
Sum of 4 skinfolds	0.98 (0.97–1.00)	0.005	0.98 (0.96–1.00)	0.111
FBG (Low blood glucose)	Reference			
Normal	0.78 (0.25–2.44)	0.671	1.91 (0.21–17.43)	0.565
Pre-diabetic	0.54 (0.16–1.65)	0.263	1.50 (0.16–14.33)	0.724
Diabetic	1.20 (0.31–4.68)	0.793	5.00 (0.46–54.30)	0.186

Abbreviations: WtHR: Waist-to-Height-Ratio; TCHOL: Total Cholesterol; HDL-C: High Density Lipoprotein Cholesterol; LDL-C: Low Density Lipoprotein Cholesterol; BMI: Body Mass Index; WC: Waist Circumference; TG: Triglyceride. WC and WtHR were not entered simultaneously due to collinearity; WC was retained and WtHR was excluded, and vice versa. Adjusted model included: age group, physical activity, BMI category, WC, blood pressure category, HDL-C, LDL-C, TG, TCHOL, LDL/HDL-C ratio, TCHOL/HDL-C ratio, sum of 4 skinfolds, and FBG.

## Data Availability

The data presented in this study are available on request from the corresponding author due to ethical restrictions.
